# Allergy to Peanuts imPacting Emotions And Life (APPEAL): the impact of peanut allergy on children, adolescents, adults and caregivers in France

**DOI:** 10.1186/s13223-020-00481-7

**Published:** 2020-10-07

**Authors:** Pascale Couratier, Romain Montagne, Sarah Acaster, Katy Gallop, Ram Patel, Andrea Vereda, Guillaume Pouessel

**Affiliations:** 1 Association Française pour la Prévention des Allergies (AFPRAL), 4, place Louis Armand - La Tour de l’Horloge, 75012 Paris, France; 2 Aimmune Therapeutics, 10 Eastbourne Terrace, London, W2 6LG UK; 3 Acaster Lloyd Consulting, Ltd, 16 Woburn Pl, Bloomsbury, London, WC1H 0BS UK; 4 Brainsell Ltd, 4 Duke Street, Richmond, TW9 1HP UK; 5 Department of Pediatrics, Children’s Hospital, F-59056 Roubaix, France; 6 Pediatric Pulmonology and Allergy Department, CHU Lille, Lille University, F-59000 Lille, France; 7 Unité de pneumologie et allergologie pédiatriques, Hôpital Jeanne de Flandre, CHRU Lille et Université Lille2, 2 Avenue Oscar Lambret, 59037 Lille, France

**Keywords:** Peanut allergy, France, Clinical history, Diagnosis, Burden, Quality of life

## Abstract

**Background:**

Peanut allergy (PA) has increased in developed countries and can have a dramatic effect on quality of life but data surrounding this is limited in France. Allergy to Peanuts imPacting Emotions And Life study (APPEAL) investigated the experience and impact of living with PA in France.

**Methods:**

Respondents affected by PA directly (children aged 8–12 years, teenagers aged 13–17 years, or adults aged ≥ 18 years) or indirectly (caregiver) completed either an online questionnaire (APPEAL-1, N = 198), or provided in-depth interviews (APPEAL-2, N = 32). Quantitative data was evaluated using descriptive statistics. Qualitative data was analysed thematically, using MAXQDA software.

**Results:**

Of 198 responders in APPEAL-1, 88% stated that PA affects their daily activities, and 74% felt isolated as a result of living with PA. Feelings of worry about exposure to peanuts on social occasions where food is involved was reported by 91%. A total of 44% reported some restrictions in their job options, 85% in socializing. Psychological impact of PA included responders feeling emotions of frustration (89%), uncertainty (87%), and stress (93%) and 93% reporting encountering instances of feeling different due to their PA. Main factors that drove PA impact included social activities and relationships; whereas main coping strategies to avoid peanuts included monitoring, communication and planning.

**Conclusion:**

The analysis of French respondents from the APPEAL study demonstrates the impact and burden of PA on allergic children, teenagers, adults and their caregivers, and highlights the unmet need to be addressed.

## Background

Peanut allergy (PA) is one of the most common food allergies in Europe, previously estimated to have a prevalence of 0.65% in the French population [1]; however, prevalence continues to rise in Europe [2, 3]. The MIRABEL survey, a large observational study conducted mainly in France including 785 children with PA [4], found that over a third of these patients also have tree nut sensitization or allergy and almost all PA patients (95%) have allergic comorbidities, with asthma (59%) and atopic dermatitis (66%) being the most common.

The current management of PA in France relies on strict avoidance of peanut and food containing peanuts, the prescription of emergency kits including adrenaline autoinjectors (AAIs) in at-risk patients, and the use of AAI in case of anaphylaxis following accidental exposure. In addition, in France, management strategies to improve the acceptance of children with food allergy and reduce the risk of anaphylaxis at school through individual healthcare plans were introduced into law in 2003 [5].

Data from the EuroPrevall project and other studies have consistently found that food allergy has a strong negative impact on the health-related quality of life (HRQL) of individuals with food allergy and their families and caregivers [6–8]. A survey of children, adolescents, and adults with food allergy in the Netherlands found that food allergic patients had poorer HRQL than the general population and that the negative impact of food allergy on HRQL was greater than that seen with diabetes [9]. Research focused specifically on PA has also found that PA can have a greater negative impact on the HRQL of affected individuals/caregivers compared to those suffering from diabetes or rheumatological diseases [10, 11]. Children with PA had significantly poorer physical HRQL, HRQL within school, and general HRQL than their non-PA siblings [12]. A US study reported significantly lower emotional functioning among children with PA, as assessed by their parents, compared with population norms; a subset of parents also reported high levels of parenting stress and child anxiety [13]. A French study has identified several factors that may be associated with poorer HRQL in PA, including experience of previous severe reactions, having family members also affected by allergies, being female, having atopic comorbidities, and having multiple food allergies [14]. Of note, the MIRABEL survey found no relationship between PA-related anxiety scores (n = 401) and the severity of previous reactions [4], which may indicate that a history of severe reactions contributes to poor HRQL by a mechanism other than increased anxiety. The MIRABEL survey also found that anxiety scores (n = 401) were higher for peanut allergic patients with atopic dermatitis (*P *= 0.003), for those with atopic dermatitis and asthma (*P *= 0.032), and for those who had received strict avoidance advice (*P *< 0.001). No significant associations were found between anxiety scores and age at diagnosis or visit, severity of the initial reaction to peanut, or eliciting doses at oral food challenge or in real life [4].

The APPEAL (Allergy to Peanuts imPacting Emotions And Life) study was conducted to assess the impact of living with PA on children, adolescents and adults with PA, as well as their caregivers. The APPEAL study was conducted in two phases (quantitative and qualitative) across eight European countries. Given the lack of research describing the impact of PA on affected individuals and their caregivers in France, this article specifically focuses on the data collected during the APPEAL study, which provides a quantitative and qualitative description of the experience and impact of PA on those members of the French population.

## Methods

### Study design

The APPEAL study was a two-stage, mixed methods (quantitative survey and qualitative interviews), cross-sectional study of the psychosocial burden of PA, conducted in eight European countries (Germany, UK, France, Spain, Denmark, Ireland, Italy and the Netherlands) [15–17]. Details of the full APPEAL study have been previously reported [15–17]. This article reports the results from participants in France only.

### Procedures

The first stage of the study (APPEAL-1) consisted of an online survey designed to assess the burden and impact of PA on individuals with PA and their caregivers (see Additional file 1: Appendix S1). As the many topics planned for this study were outside the scope of existing quality-of-life measures, a novel, study-specific instrument was developed to address unmet research needs regarding the impact of PA. The APPEAL survey was developed by the APPEAL advisory board, which included representatives of eight patient advocacy groups (one from each of the eight countries the study was conducted in) and a specialist panel of five healthcare professionals and research specialists. The questionnaire was developed through an iterative process that included online plot testing with revisions made according to respondent feedback. The survey was designed to assess the burden and impact of PA on individuals with PA and their caregivers. For most survey questions, a five-point response scale was used (in general, 1 indicated the lowest impact and 5 highest impact). The questionnaire included demographics and clinical characteristics, practical issues of PA management and psychosocial impacts.

The second stage (APPEAL-2) consisted of in-depth telephone or in-person interviews conducted by a native French speaker. All interviews with children were conducted in-person; parents/caregivers were not present during child interviews and vice versa. The interview guide allowed participants to spontaneously describe how PA affects them in addition to using pre-specified probes if concepts were not raised. Caregivers of children and teenagers (aged 4 to 17 years) with a diagnosis of PA were asked about the impact of PA on their child with PA as well as on their own life. All interviews were recorded and transcribed into English.

### Participants

Two independent samples of participants were recruited for APPEAL-1 and APPEAL-2. APPEAL-1 participants were recruited through patient advocacy groups and specialist patient recruitment panels using various methods, such as announcements on websites and direct mail contact; APPEAL-2 participants were all recruited through specialist patient recruitment panels that engaged participants drafted from databases of individuals willing to participate in research studies.

Participants were eligible for APPEAL-1 if they were adults with a self-reported diagnosis of PA or a caregiver to an individual of any age with a diagnosis of PA, a resident of France and had not taken part in a market research study on PA in the previous 2 months.

Participants were eligible for APPEAL-2 if they were a child (aged 8–12 years), teenager (aged 13–17 years) or adult (aged 18–30 years) with self-reported moderate or severe PA or a caregiver of a child aged 4–17 years with moderate or severe PA, and resident of France. Participants were excluded from APPEAL-2 if they had never experienced a reaction to peanut in their day-to-day life (e.g. only as a result of a food challenge) and recruitment aimed for a minimum of 50% with a self-reported severe PA and at least 25% who had experienced a life-threatening reaction (defined as requiring intubation or intravenous adrenaline) or used an AAI.

Each stage of the study was reviewed and approved by an independent ethics board (APPEAL-1: Freiburger Ethik-Kommission International; APPEAL-2: Western Independent Review Board). Participants were provided with information about the study and gave informed consent prior to taking part in either stage.

### Analysis

APPEAL-1 data were analysed using descriptive statistics. The APPEAL-1 data were analysed for the sample as a whole, with self-reported and proxy-reported data combined. APPEAL-2 demographic and background data were analysed using descriptive statistics. APPEAL-2 data were analysed using thematic analysis. This involved a team of analysts coding the qualitative text of the transcripts using a coding frame. APPEAL-2 analysis was assisted by MAXQDA, a qualitative software tool. Saturation, the point at which no new information is obtained from additional qualitative data, was assessed using saturation tables [18]. A conceptual model, which is a visual representation of the themes and possible relationships between themes, was developed using the concepts identified during the analysis.

## Results

### Study participants

A total of 198 adults in France completed the APPEAL-1 survey between 10 November and 11 December 2017, including 60 adults with PA and 138 caregivers (102 parents/36 non-parents). The 138 caregivers (40 caregivers for adults, 37 for teenagers and 61 for children) provided self-reported data on the impact on themselves; 80 also provided proxy-reported data on the person with PA they care for.

A total of 32 individuals from France participated in APPEAL-2 (8 adults with PA, 8 teenagers with PA, 8 children with PA and 8 caregivers of a child with PA). Caregiver participants in APPEAL-2 were asked about the impact of PA on the individual they cared for and about the impact of PA on themselves as a caregiver. A demographic summary is shown in Table 1. Most of the age groups contain both males and females, however, all caregiver participants in APPEAL-2 were female. In most groups over half of participants were prescribed an AAI.

**Table 1:** APPEAL-1 and APPEAL-2 sample characteristics

Characteristic	APPEAL-1				APPEAL-2			
	Adults (self- and proxy-report)	Children (proxy-report) Age 0–3 years	Children (proxy-report) Age 4–12 years	Teenagers (proxy-report) Age 13–17 years	Caregivers	Adults	Children (Age 8–12 years)	Teenagers
N	100	6	55	37	8	8	8	8
Age: Mean (SD), years	35.9 (16.2)	1.7 (1.0)	7.9 (2.5)	14.9 (1.3)	37.4 (4.4)	24.4 (3.0)	10.0 (1.4)	15.1 (1.0)
Gender: Female, n (%)	67 (67)	2 (33)	29 (53)	17 (46)	8 (100)	6 (75)	3 (38)	5 (63)
Other FA, n (%)								
Tree nuts	51 (51)	4 (67)	42 (76)	27 (73)	2 (25)^a^	0 (0)	1 (13)	1 (13)
Other food	69 (69)	4 (67)	48 (87)	29 (78)	2 (25)	4 (50)	4 (50)	4 (50)
AAI prescribed: Yes, n (%)	47 (47)	5 (83)	40 (73)	23 (62)	5 (63)^a^	7 (88)	4 (50)	7 (88)

*AAI* adrenaline autoinjector, *FA* food allergy, *SD* standard deviation

^a^ Child’s FA/AAI prescription

### APPEAL-1

**Fig. 1:**
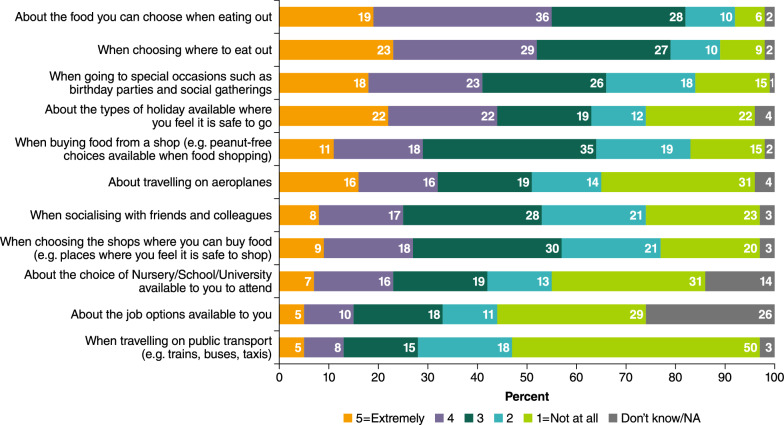
Restrictions on choice in different situations. *NA* not applicable

The APPEAL-1 survey assessed whether participants felt their choices were restricted by PA in different contexts, using a scale from “1 = not at all” to “5 = extremely” restricted and “don’t know/not applicable”. As shown in Fig. 1, most participants reported that they felt at least a little restricted (rating ≥ 2) in choosing: where to eat out (89%, including 52% “very” or “extremely” restricted); food options when eating out (93%, with 55% “very” or “extremely”); buying food (83%, with 29% “very” or “extremely”) and shops where they can buy food (78%, with 27% “very” or “extremely”). Furthermore, some participants reported that they felt restricted in choices *not* directly related to food, such as choice of schools (55% rating ≥ 2), job options (44%), socialising (74%), and most also felt restricted by PA when going to special occasions (85%). Most participants (88%) said that PA impacted their daily activities, with 85% reporting that extra planning was needed for managing PA for daily activities and special activities such as holidays (87%). When asked to rate their HRQL because of having to make extra plans, on a scale of 1 to 5 (where 1 = excellent and 5 = poor), two-thirds of participants (67%) gave their HRQL a rating of 2 or 3, although 13% rated it as excellent, and only 3% rated it as poor.

**Fig. 2:**
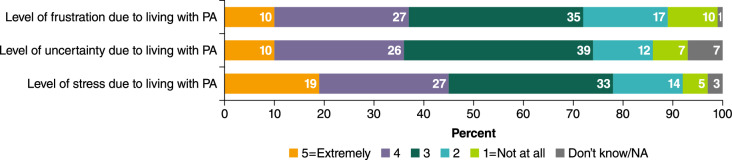
Levels of frustration, uncertainty and stress of living with PA. *NA* not applicable, *PA* peanut allergy

APPEAL-1 participants were also asked about the psychosocial impact of PA. Figure 2 shows that 89% experienced some level of frustration (score of ≥ 2 on a scale of “1 = not at all” to “5 = extremely frustrated”) due to living with PA; 10% reporting feeling “extremely frustrated”. In addition, 87% reported at least some uncertainty related to living with PA and almost all (93%) reported feeling stress; a third reported scores of 4 or 5 for each outcome.

**Fig. 3:**
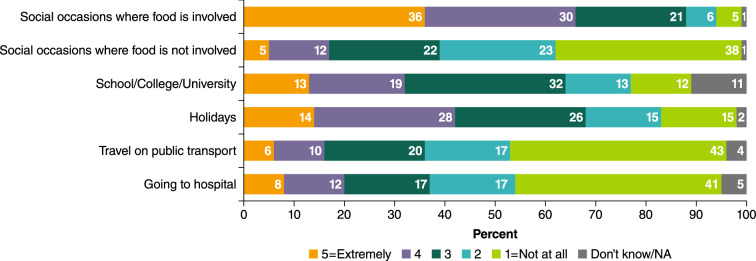
Level of worry in different situations. *NA* not applicable

Almost all participants (93%) said they worry about exposure to peanut on social occasions where food is involved (score of ≥ 2 on scale from “1 = not at all” to “5 = extremely worried”), and nearly two-thirds (62%) reported worry about exposure during occasions where food is not involved (Fig. 3). Many participants also reported that they worry about exposure to peanut in multiple other settings including at school/college/university (77%), on holiday (83%), on public transport (53%) and at hospital (54%). Many participants reported signs of anxiety; over a third (37%) said that they frequently felt anxious, 31% said they frequently felt tense, almost a third (29%) said they rarely felt calm, and 55% indicated they often feel that something bad will happen.

**Fig. 4:**
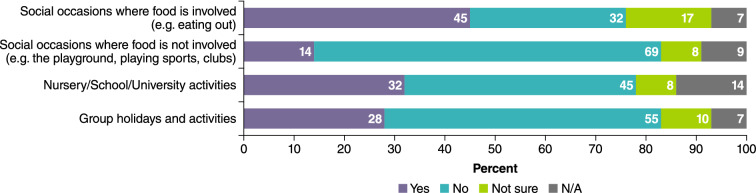
Excluded from different situations due to PA. *NA* not applicable

More than fourout of ten (43%) APPEAL-1 participants had experienced bullying due to their PA. Of these, 23% reported experiencing bullying frequently or very frequently, and 40% described the impact of the bullying as severe. Most participants (86%) reported that they have been made to feel different because of their PA, and half (50%) reported this happens either quite or very frequently. As shown in Fig. 4, nearly half of participants (45%) have been excluded from social situations involving food; however, only 14% have been excluded from social situations not involving food. Approximately a third have been excluded from nursery/school/university activities (32%) and group holidays or activities (28%). Furthermore, three-quarters (74%) have felt isolated as a result of living with PA, yet only 26% reported that this occurs very or quite frequently.

When asked about how they cope with their PA or their child’s PA now on a scale of 1 (extremely well) to 5 (not at all well), nearly three-quarters (71%) of participants responded with a rating of either 1 or 2; however, when asked about coping when they were diagnosed, only 27% responded with a rating of 1 or 2, and 18% rated their coping at diagnosis as 5 (not at all well). Of the participants who had been prescribed an AAI (n = 115), a mean of 11 min training in how to use it was provided and only a third were satisfied with the training they had received (1 or 2 on a 5-point scale, from completely satisfied to not at all satisfied). Among these participants, only half (49%) were confident in knowing *when* to use an AI, and less than two-thirds (60%) were confident in knowing *how* to use it.

### APPEAL-2

The qualitative data from APPEAL-2 found that participants use three types of coping strategies to avoid peanut: daily monitoring/vigilance, communication and practicalities/planning. Daily monitoring included checking ingredients, staying away from people eating peanut and hygiene practices such as frequent handwashing. Communication involved having to inform others, such as restaurant staff, about their PA. Some children and adolescents were reluctant to disclose their PA to others due to embarrassment. Practicalities and planning included buying and preparing food, carrying emergency medication such as an AAI, and planning ahead to ensure suitable food would be available at social events or activities. Table 2 shows some of the quotations illustrating these coping strategies.

**Table 2:** APPEAL-2: sample quotes from French APPEAL-2 participants

	Sample quotes from adults, adolescents and children with PA	Sample quotes from caregivers
Daily coping strategies		
Daily monitoring/vigilance	“If we find a food that we often use, even if we use it all the time we still check if it’s peanut or not because they may not use peanuts and suddenly, they use peanuts”—Female, age 10	“We have completely banished peanuts from the house, and whenever we are around other people, we are extremely careful.”—Female caregiver of boy aged 5
Communicating	“I don’t care, but I don’t like people in general to know. For me, it’s something private that doesn’t have to be known.”—Male, age 15	“Whenever she is invited to a birthday party, I warn the mom or the person organising the party when I drop her. Then they are happy I mentioned it and they tell me what they had planned for snacks.”—Female caregiver of girl aged 9
Practicalities	“I always choose vacation spots that are near to the hospital… it has to be less than half an hour from a hospital or there are too many risks. Then I call the hotel when it’s all-inclusive to know if they cook with groundnut oil.”—Female, age 27	“I ask them to be really specific on what they’re going to make and which brand they’re going to use. It’s very intrusive, it’s like we’re the FBI or something.”—Female caregiver of girl aged 15
HRQL impacts		
Social/school activities	“For parties, it depends if it’s a close friend, he’ll be more careful what he takes, he won’t put peanuts on the tables. With the others, it’s more complicated, maybe I’ll eat before I go, so I don’t eat what’s there, to limit the risks. I will warn them, but then they won’t necessarily be careful.”—Female, age 15	“It was a bit hard on a social level at first, but our friends understood pretty quickly. It’s in moments like this where we get to see who our real friends are. Sometimes, we are told that we should come without children because it is complicated.”—Female caregiver of girl aged 15
Relationships	“Some of my friends find it weird that I cannot eat the same things as them. So I tell them, but it’s useless because they don’t understand. They only understand that they should stay away from me.”—Male, age 9	“I feel like my daughter sees me as a tyrant and not as a cool mom compared to her friends’ moms.”—Female caregiver of girl aged 8
Emotions	“It pains me because I’m the only one with an allergy and when I’m invited to a party, everyone can eat without thinking and I cannot.”—Male, age 10	“I felt stressed out, and anxious, but now I simply feel a bit annoyed with the fact that we need to be careful about what she eats.”—Female caregiver of girl aged 12
Bullying	“There were people eating chocolate at school and they offered some to me, and I said no because I was allergic. And then, they started laughing at me because they could eat it and I couldn’t. It only happened a few times, but it makes me feel bad. I always tell my mom and she talks to the kids’ parents to make them stop.”—Male, age 9	“So the opinion of others really had an impact on him as well back then, because certain kids can be quite insensitive when they learn he is allergic, as if it were a disease.”—Female caregiver of boy aged 12
Work	“When I see my colleagues going to the fast-food restaurant next door and I eat from my lunchbox, I am frustrated. I tell myself that I could have shared a good time with my colleagues and I am there with my lunchbox.”—Female, age 28	“So I had to switch jobs because of that and this is why I can talk to you at this time. I cannot have regular office hours because I’m too scared he might have a reaction so I don’t want him to eat in the canteen.”—Female caregiver of boy aged 9

*HRQL* health-related quality of life

**Fig. 5:**
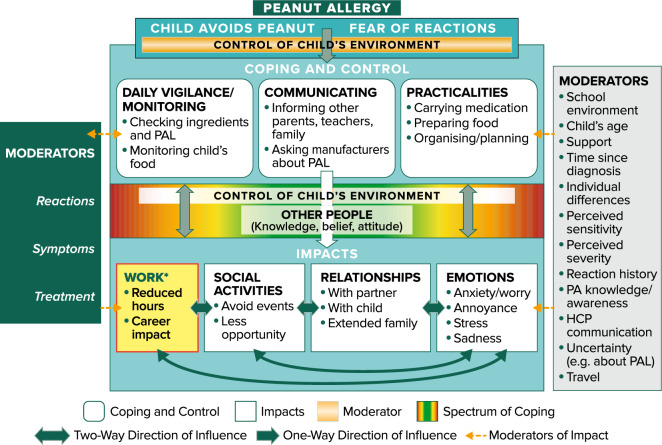
APPEAL-2 conceptual model. The arrows indicate the direction of influence. The colour spectrum between “Coping and Control” and “Impacts” represents the range of reported behaviours from a highly vigilant approach to a careless approach (both in red)—each of which can have a negative impact—with the middle section indicating a more positive or neutral impact. Green indicates a balanced approach and a positive impact. *HCP* health care professional, *PA* peanut allergy, *PAL* peanut allergy labels. *Indicates impact on adults with PA and caregivers only

Avoiding peanut and the coping strategies used to ensure avoidance impacted various areas of participants’ lives, in particular social and school activities, relationships, emotional functioning and—for adults and caregivers only—work. Table 2 shows example quotations from participants illustrating these concepts. The relationships between impacts are illustrated in the conceptual model in Fig. 5. Most participants reported negative impacts of PA on their social activities, including not attending parties if peanut would be served, avoiding certain places such as cinemas, and having limited food options when attending social events.

Most participants reported that PA and the coping strategies required impacted their relationships. For some there were negative impacts on relationships with their partner as they also had to avoid peanut; others reported being excluded from activities with friends because of their PA. A small number of participants (1 adult, 1 child and 3 caregivers) had experienced some bullying or teasing due to their PA; no adolescents reported any PA-related bullying. Of note, in the international APPEAL-2 study, bullying was reported by teenage participants with PA only after prompting, possibly owing to the sensitivity of the topic [17]. As was observed in the international cohort, among French participants a lower rate of bullying was observed in APPEAL-2 compared with APPEAL-1, possibly due to reluctance of participants to discuss bullying during in-person interviews or lack of direct child/adolescent reporting in APPEAL-1.

Almost all participants reported an emotional impact of PA. The most common emotions associated with PA were anxiety, worry and/or fear related to experiencing a reaction. Caregivers reported increased anxiety when they do not have control over their child’s food or environment, in addition to the constant underlying anxiety reported by some caregivers. Other commonly reported emotional impacts included feeling different from others and feeling annoyance or frustration.

A small number of adults with PA and caregiver participants reported an impact of PA on their work or career. Two caregivers had reduced their working hours to allow them more time for food preparation and management of their child’s PA. Adult participants with PA reported some negative impacts of PA on their work, such as avoiding going out to eat with colleagues.

The interview results also identified two key moderators that can have positive or negative impacts on participants’ HRQL: control (over food and environment) and other people. The conceptual model shown in Fig. 5 illustrates the relationships between the main coping strategies and their impacts. Moderators such as allergic reactions, symptoms, and treatment may have a bidirectional relationship with coping and control and may also impact social/school/work activities, relationships, and emotions.

Table 3 shows three example case studies from the APPEAL-2 sample. The profiles summarise the demographics, self-reported severity, level of confidence in managing PA and control of PA, AAI possession, reaction history, and the main impacts reported in the interview for participants who reported minimal impacts, moderate impacts, and severe impacts of PA on their HRQL. These examples demonstrate that people’s reported levels of control and confidence with PA may not adequately reflect the impact PA has on their lives and highlight the value of capturing impacts in order to better understand any potential unmet need.

**Table 3:** Case studies outlining 3 participants, each reporting minimal, moderate or severe impact from APPEAL-2

	Minimal impact	Moderate impact	Severe impact
Demographics	Male, age 15	Male, age 23	Male, age 9 (child report)
Severity	Moderate	Severe	Moderate
AAI?	Yes	Yes	No
Confidence^a^	Confident	Quite confident	Confident^b^
Control^a^	Good	Good	None or very little^b^
Reaction history	Had two mild reactions when 8 years old (before diagnosis)	Very rarely has reactions, last one was 6 months ago. He went to hospital, received an infusion and adrenaline injection	One reaction aged 4 or 5 years (before diagnosis), experienced breathing difficulties and went to hospital
Main impacts	He avoids foods that he knows contain peanuts, but it has minimal impact	He is very cautious, always carries emergency medication He doesn’t eat food in a restaurant if there is doubt about whether it contains peanuts; generally has good control PA does not affect his daily activities; he is just careful	Not allowed to go to birthday parties He is not allowed to eat at restaurants Tells others not to come near him after eating peanuts or to wash their hands Parents keep him away from peanuts; he has to go upstairs when people are eating peanuts at home Children at school laughed at him because of his PA

*PA* peanut allergy

^a^How confident do you feel in managing reactions to peanut; how much control do you have over your peanut allergy?

^b^Caregiver report of how confident and how much control they believe the child feels

## Discussion

The APPEAL study was a large pan-European study exploring the psychosocial burden of PA in Europe. Results reported here focus on the experience of people with PA in France and their caregivers, highlighting the psychosocial burden of PA in day-to-day life. APPEAL-1 results show the high proportion of people in France reporting psychosocial burden, including feeling restricted in their choices and excluded from activities because of their PA, as well as feeling the emotional impacts of frustration, uncertainty, stress, and anxiety caused by living with PA. The results also showed nearly half of participants had experienced bullying due to their PA, and that nearly half of those felt the impact of the bullying was severe.

The qualitative data collected in APPEAL-2 add detail to the findings of APPEAL-1, demonstrating wide variation in the levels of impact, and through the conceptual model, illustrating the range of coping strategies and moderators and their subsequent impact on different areas of participants’ lives. As detailed in the primary APPEAL-2 paper, previous qualitative research in families of children with PA has noted similar PA-specific impacts as the current study, such as feeling excluded, the social challenges of eating in restaurants, and the lack of public awareness of PA [17]. Previous studies of PA and/or food allergy have also identified some of the same moderating factors as the caregiver conceptual model of APPEAL-2, such as the burden of meal preparation, ensuring the home is nut-free, and barriers to teenagers’ transitions to independence [17]. The APPEAL-2 results provide further evidence that although some people with PA manage well with minimal HRQL impact; for some there is an unmet need for a treatment that reduces the impact of PA on their life.

The results of the APPEAL study are in line with previous studies that documented the negative impact of PA on HRQL [19], and indicate that findings from research conducted in other countries—including the UK [12, 20], US [13, 21, 22], Thailand [23], Denmark [24], and Canada [22, 25]—also apply to individuals affected by PA in France. In addition, these results, particularly from APPEAL-2, support the previous finding that there is a large variation in the impact of PA [13], with many individuals coping well while some report a significant psychosocial burden. The impact of PA on HRQL may vary depending on the management strategies used by children with PA and their families [26]. The results also support previous findings in food allergy that a third of children with food allergy report having been bullied specifically because of their food allergy [27].

The quantitative and qualitative methods used in this study provide evidence of the psychosocial burden of PA in France specifically. For example, the survey data showed that most participants report an impact of PA on their daily activities; the qualitative data provided details of the specific ways in which daily lives are impacted (such as in coping strategies to avoid peanuts and the practicalities of being prepared in case of a reaction). Furthermore, the survey data showed various psychosocial impacts experienced by participants, and similar impacts were reported spontaneously by participants in the qualitative interviews therefore providing support for the quantitative findings. The survey data also showed that despite a high proportion of participants reporting each of the psychosocial impacts, most participants reported coping well with their PA at the time of the survey. In addition, the APPEAL-1 data revealed that most participants who had been prescribed an AAI were not satisfied with the amount of training they had received in how to use it, nor were many participants confident in knowing when and how to use an AAI. These results suggest that improvements could be made in terms of AAI training for individuals with PA and their caregivers.

The qualitative data highlighted in the case studies show that although someone reports confidence in managing their PA, it does not necessarily mean PA has a minimal impact on their daily life. The case studies also suggest that despite having experienced a severe reaction, not every child is prescribed an AAI. The qualitative data help to explain and add depth to the survey findings and highlight the complex relationship between the daily coping/control behaviours and the impact on HRQL.

Other people and their awareness of and attitude towards PA are central moderators in the conceptual model, indicating the important role that other people and society have to play in the impacts of PA on individual’s lives. The high prevalence of bullying reported in APPEAL-1, and the well-documented association of bullying with decreased QoL and increased distress in children with food allergy [27–29], also suggest that the wider population needs educating as to the serious nature of PA and the potential impacts it has on individuals.

Some limitations should be considered when interpreting the results of this study. First, although APPEAL-2 included interviews with children and adolescents, APPEAL-1 relied on proxy-reporting of the impact of PA on children and adolescents; accordingly, the views of children and adolescents with PA were not captured directly in APPEAL-1. Clinical severity of PA was a subjective measure based on self- or proxy-assessment, which was recommended as appropriate by clinicians reviewing the study protocol, and no objective measures were required to confirm PA, although most reported that the PA diagnosis was confirmed using objective measures (peanut-specific IgE test, peanut skin-prick test, and/or oral food challenge) [15]. Although recruitment aimed for diversity in sex, minimum quotas were not set. In addition, as socioeconomic data were not collected, it is not possible to assess the impact of socioeconomic factors on study results.

Some previous studies in food allergy have found that parents reported significantly better HRQL for their child than the children themselves reported [24, 30, 31]; therefore, the results may have underestimated the psychosocial burden for children and adolescents. Recruitment through patient advocacy groups also may have introduced some selection bias in APPEAL-1—with more motivated and potentially more severely affected individuals who are members of a support group recruited—however, using various recruitment methods minimised the potential for bias. Future research could apply the conceptual model developed from qualitative data to develop hypotheses to explore using the quantitative data, for example, using structured equation modelling.

## Conclusions

This large survey and interview study highlights the psychosocial burden of PA in France for adults, adolescents, children and caregivers. The study demonstrates the wide variation in the level of impact of PA and the unmet need for those who report a significant impact. Providers should be aware that some patients with PA and their caregivers appear to be negatively impacted to a greater degree than others and should be prepared to explore these issues. Additionally, there is a need for future research on the impact of interventions for PA on the HRQL of patients and their caregivers.

## Supplementary information

**Additional file 1.** APPEAL Questionnaire.

## Data Availability

All data generated or analysed during this study are also available from the corresponding author on reasonable request.

## Abbreviations

AAI: Adrenaline autoinjector
APPEAL: Allergy to Peanuts imPacting Emotions And Life study
HRQL: Health-related quality of life
PA: Peanut allergy
