# Supplementary material for: Allergy to Peanuts imPacting Emotions And Life (APPEAL): the impact of peanut allergy on children, adolescents, adults and caregivers in France
Source: Allergy Asthma Clin Immunol. 2020 Oct 7;16:86. doi: 10.1186/s13223-020-00481-7 (PMC7541331; doi:10.1186/s13223-020-00481-7)
Supplement: Supplementary file 1 — Additional file 1. APPEAL Questionnaire. [file 13223_2020_481_MOESM1_ESM.pdf]

brainsell
intelligent research
undeniable truth

APPEAL

Allergy to Peanuts imPacting Emotions And Life

QUESTIONNAIRE

FOR A

QUALITY OF LIFE STUDY INTO BURDEN OF LIVING WITH

A PEANUT ALLERGY

brainsell
intelligent research
undeniable truth

APPEAL

There will now follow a few questions that are designed to quickly assess whether the main questionnaire is appropriate for you.

Please note you may leave the survey at any time and continue from the point you reached by clicking the link beneath the progress bar.

Screen out/quota full text

Thank you for your interest in this survey. Unfortunately, your responses indicate this survey would not be appropriate for you. We thank you for your willingness to participate in this survey.

If screened out on S2: Thank you for your interest in this survey. Unfortunately, your responses indicate you are too young to participate. However, your parents/guardians can participate by registering their interest on (PAG website)

Initial questions

S1. Please select the country you live in:

A	Denmark	
B	France	
C	Germany	
D	Italy	
E	Ireland	
F	Netherlands	
G	Spain	
H	UK	
I	Other country within Europe (please specify ___________)	
J	Other country outside of Europe (please specify ___________)	Screen out

S2. How old were you on your last birthday?

A	18 or older	
B	Less than 18	Screen out

S3. Do you have peanut allergy?

A	Yes	
B	No	

If Yes in S3

S3.1 Are you in a clinical trial for a medicine to treat your peanut allergy?

A	Yes	
B	No	

brainsell
intelligent research
undeniable truth

S4. How many other adults (18+ years) and children (<18 years) in your household have peanut allergy? Please do not include yourself

A	_____ ADULTS with peanut allergy	

B	_____ CHILDREN with peanut allergy	

If Yes in S3 and sum of adults and children in S4=0, go to S7 and code S5 as A: the person with peanut allergy

Ask only if [No in S3 and sum of adults and children in S4=0

S4.1 Do you regularly look after any family or friends with peanut allergy

A	Yes	
B	No	Screen out

If Yes in S3 and the sum of adults and children in S4 > or = 1

The remainder of the questions need to be answered based on only ONE individual in your household who has peanut allergy. Please note, you will also be able to take the questionnaire again if you wish to respond on behalf of another person with peanut allergy. Instructions for this will follow at the end.

S5. Please indicate how you would like to answer the remainder of the questions.
I would like to answer the remainder of the questions…

A	based on myself as the person with peanut allergy	Go to S7
B	as the parent carer of a person with peanut allergy	
C	as a non-parent carer of a person with peanut allergy	

OR

If No in S3, and the sum of adults and children in S4 =1

For the remainder of the questions, please base your answers on the person with peanut allergy in your household

S5.1 Please indicate how you would like to answer the remainder of the questions.
I would like to answer the remainder of the questions…

B	as the parent carer of the person with peanut allergy	
C	as a non-parent carer of the person with peanut allergy	

OR

If No in S3 **and** the sum of adults and children in S4 >1, **or** Yes for S4.1

The remainder of the questions need to be answered based on only ONE individual who has peanut allergy

S5.2 Please indicate how you would like to answer the remainder of the questions.
I would like to answer the remainder of the questions…

B	as the parent carer of a person with peanut allergy	
C	as a non-parent carer of a person with peanut allergy	

If B or C in S5/S5.1/S5.2

S6. Is the person that you are answering about currently in a clinical trial for a medicine to treat their peanut allergy?

A	Yes	
B	No	

Ask all

S7. Please indicate [**your current age** if S5=A / **the current age of your child with peanut allergy** if S5 or S5.1 or S5.2=B] / **the person with peanut allergy who you look after** if S5 or S5.1 or S5.2=C]**:**

A	_______Free text numeric 0 to 99	
B	Prefer not to say (if S5=A)	

S8. Please indicate [**your gender** if S5=A] / **the gender of your child with peanut allergy** if S5 or S5.1 or S5.2=B] / **the person with peanut allergy who you look after** if S5 or S5.1 or S5.2=C]**:**

A	Female	
B	Male	
C	Prefer not to say (if S5=A)	

Main questionnaire

THE FOLLOWING TEXT IS ON **ALL SCREENS**

If S5=A

Please answer the remaining questions based on your own experience of living with peanut allergy

If S5 or S5.1 or S5.2=B

Please answer the remaining questions based on your experience as the parent of your [age in S7] year old child with peanut allergy.

If S5 or S5.1 or S5.2=C

Please answer the remaining questions based on your experience as the carer of the [age in S7] year old person with peanut allergy who you look after.

Poly-Allergies and other conditions

Q1. In general, how important is it for you to be able to avoid peanuts, tree nuts, and other foods to reduce the risk of an allergic reaction? Please indicate on a scale 1 to 5, where 1 is Not at all important and 5 is Extremely important.

A: Peanut specifically

Not at all important Extremely important

1-------------------------- [scale of 1 to 5] ---------------------------5

B: Foods with a trace of peanut

Not at all important Extremely important

1-------------------------- [scale of 1 to 5] ---------------------------5

C: Tree nuts (NOT peanut)

Not at all important Extremely important

1-------------------------- [scale of 1 to 5] ---------------------------5

D: Other foods

Not at all important Extremely important

1-------------------------- [scale of 1 to 5] ---------------------------5

Q2. Approximately, how many times per year would you say [you if S5=A / you take your child to if S5 or S5.1 or S5.2=B / you take the person who you look after to if S5 or S5.1 or S5.2=C] visit a healthcare professional (e.g. Allergist, Primary Care Physician, other Specialist doctor, Nurse, Dietician, Nutritionist, or Psychologist) because of the (a) Peanut allergy specifically (b) Tree Nut allergy (other nuts apart from peanut), and (c) other Food allergies. Please provide your best estimates.

A	Number of visits for Peanut allergy specifically _______	
B	Number of visits for Tree Nut allergy _______	
C	Number of visits for other Food allergies ________	

The next few questions are about other foods and nuts, NOT peanuts.

Q3. [Have you if S5=A / Has your child with peanut allergy if S5 or S5.1 or S5.2=B / Has the person who you look after with peanut allergy if S5 or S5.1 or S5.2=C] been diagnosed as having an allergy to tree nuts (NOT peanuts)?

A	Yes	
B	No	
C	Not sure	

Q4. Which of the foods listed below [have you if S5=A / has your child if S5 or S5.1 or S5.2=B / has the person who you look after if S5 or S5.1 or S5.2=C] been diagnosed as having an allergy to? Please select all foods [you if S5=A / your child if S5 or S5.1 or S5.2=B / the person who you look after if S5 or S5.1 or S5.2=C] have been diagnosed allergic to.

A	Celery	
B	Cow milk and dairy products	
C	Egg	
D	Fish	
E	Fruit	
F	Meat or poultry	
G	Mustard	
H	Peach	
I	Seeds (e.g. Poppy, Pumpkin, Sunflower)	
J	Sesame	
K	Shellfish/crustacean/molluscs	
L	Soya beans / other legumes	
M	Sulphites	
N	Wheat/gluten	
O	Other (please specify) __________	
P	None others	

Q5. Not including allergies, [do you if S5=A / does your child if S5 or S5.1 or S5.2=B / does the person who you look after if S5 or S5.1 or S5.2=C] have any long-term illness which limits your daily activities?

A	Yes	
B	No	
C	Not sure	

Q6. Which of the following conditions, if any, [have you if S5=A / has your child if S5 or S5.1 or S5.2=B / has the person who you look after if S5 or S5.1 or S5.2=C] been diagnosed with?

A	Allergic rhinitis (hay fever)	
B	Asthma / breathing disorder	
C	Diabetes Type 1	
D	Diabetes Type 2	
E	Eating disorders	
F	Gastrointestinal / digestive disorder	
G	Heart disease	
H	Mood disorders (e.g. depression)	
I	Skin disorders (e.g. eczema)	
J	Other (please specify) __________	
K	None	

Diagnosis of peanut allergy

The remaining questions are about PEANUT allergy

Q7. At what age did [you if S5=A / your child if S5 or S5.1 or S5.2=B / the person who you look after if S5 or S5.1 or S5.2=C] have [your if S5=A / their if S5 or S5.1 or S5.2=B or if S5 or S5.1 or S5.2=C] FIRST allergic reaction to PEANUT?

A	_______Free text numeric 0 to 99	
B	Never	

Q8. At what age was [your if S5=A / your child's if S5 or S5.1 or S5.2=B / the person who you look after's if S5 or S5.1 or S5.2=C] peanut allergy FIRST diagnosed by a HEALTHCARE PROFESSIONAL?

A	_______Free text numeric 0 to 99	
B	Never been diagnosed by a healthcare professional	

If Q8=B

Q9. Please briefly explain why [**you** if S5=A / **your child** if S5 or S5.1 or S5.2=B / **the person who you look after** if S5 or S5.1 or S5.2=C] [**have** if S5=A / **has** if S5 or S5.1 or S5.2=B or if S5 or S5.1 or S5.2=C] **never been diagnosed by a healthcare professional for** [**your** if S5=A / **their** if S5 or S5.1 or S5.2=B or if S5 or S5.1 or S5.2=C]
peanut allergy:

>>skip

Ask if Q8=A; do not ask if Q8=B

Q10. Thinking about the first time [**your** if S5=A / **your child's** if S5 or S5.1 or S5.2=B / **the person who you look after's** if S5 or S5.1 or S5.2=C] **peanut allergy was diagnosed, how reassured were you with the advice given? Please rate on a scale 1 to 5, where 1 is Completely reassured and 5 is Not at all reassured.**

Completely reassured Not at all reassured

1--------------------------- [scale of 1 to 5] ----------------------------5

[] I do not remember

Ask if Q8=A; do not ask if Q8=B

Q11. Which type of healthcare professional FIRST diagnosed [**your** if S5=A / **your child's** if S5 or S5.1 or S5.2=B / **the person who you look after's** if S5 or S5.1 or S5.2=C] **peanut allergy?**

A	Allergist / Allergy Specialist Doctor	
B	Immunologist / Immunology Specialist Doctor	
C	Emergency Doctor	
D	Pediatrician	
E	Primary Care / Family Doctor	
F	Allergy Nurse	
G	Other Nurse	
H	Dietician	
I	Nutritionist	
J	Other (please specify) __________	

Ask if Q8=A; do not ask if Q8=B

Q12. How has [**your** if S5=A / **your child's** if S5 or S5.1 or S5.2=B / **the person who you look after's** if S5 or S5.1 or S5.2=C] **peanut allergy been diagnosed? Please tick all that apply**

A	Clear clinical reaction to peanut (or peanut containing foods)	
B	Skin Prick Test to peanut	
C	Blood test (IgE to peanut)	
D	Food challenge to peanut in a hospital/clinic	
E	Other (please specify) __________	
F	I do not remember	

brainsell
intelligent research
undeniable truth

Ask all

Q13. Which of these healthcare professionals [have you if S5=A / has your child if S5 or S5.1 or S5.2=B / has the person who you look after if S5 or S5.1 or S5.2=C] EVER seen for [your if S5=A / their if S5 or S5.1 or S5.2=B or if S5 or S5.1 or S5.2=C] peanut allergy? Please select all of the types of healthcare professionals [you if S5=A / your child if S5 or S5.1 or S5.2=B / the person who you look after if S5 or S5.1 or S5.2=C] [have if S5=A / has if S5 or S5.1 or S5.2=B or if S5 or S5.1 or S5.2=C] seen for [your if S5=A / their if S5 or S5.1 or S5.2=B or if S5 or S5.1 or S5.2=C] peanut allergy since it was first diagnosed.

A	Allergist / Allergy Specialist Doctor	
B	Immunologist / Immunology Specialist Doctor	
C	Emergency Doctor	
D	Pediatrician	
E	Primary Care / Family Doctor	
F	Allergy Nurse	
G	Other Nurse	
H	Dietician	
I	Nutritionist	
J	Psychologist	
K	Psychotherapist	
L	Other (please specify) __________	

Q14. When did you last see a healthcare professional about [your if S5=A / your child's if S5 or S5.1 or S5.2=B / the person who you look after's if S5 or S5.1 or S5.2=C] peanut allergy?

A	Less than 6 months ago	
B	In the last 6 – 12 months	
C	In the last 1 – 2 years	
D	In the last 2 to 5 years	
E	More than 5 years ago	
F	Never	

Worst (Most Severe) Allergic Reaction

Skip this section (Q15 to Q19) if Yes in S3.1 or S6

Q15. How long ago was [your if S5=A / your child's if S5 or S5.1 or S5.2=B / the person who you look after's if S5 or S5.1 or S5.2=C] WORST allergic reaction (a) specifically to peanut and (b) to any other food?

		Peanut	Any other food
A	Less than 6 months ago		
B	In the last 6 – 12 months		
C	In the last 1 – 2 years		
D	In the last 2 to 5 years		
E	More than 5 years ago		
F	Not sure		
G	Never had an allergic reaction		

Now we would like you to think just about [your if S5=A / your child's if S5 or S5.1 or S5.2=B / the person who you look after's if S5 or S5.1 or S5.2=C] WORST allergic reaction specifically to PEANUT.

Ask if Q15=A to F

Q16. Now please just think about [your if S5=A / your child's if S5 or S5.1 or S5.2=B / the person who you look after's if S5 or S5.1 or S5.2=C] worst allergic reaction to peanut. Would you describe this reaction as mild, moderate, or severe?

A	Mild	
B	Moderate	
C	Severe	
D	Not sure	

Ask if Q15=A to F

Q17. Was hospitalisation and/or emergency medication e.g. an adrenaline/epinephrine autoinjector pen or an antihistamine required for [your if S5=A / your child's if S5 or S5.1 or S5.2=B / the person who you look after's if S5 or S5.1 or S5.2=C] worst allergic reaction to peanut?

A	Yes, both hospitalisation and emergency medication	
B	Yes, hospitalisation only	
C	Yes, emergency medication only	
D	No, neither hospitalisation nor emergency medication	
E	Do not remember	

Ask if Q15=A to F

Q18. What were the main symptoms of [your if S5=A / your child's if S5 or S5.1 or S5.2=B / the person who you look after's if S5 or S5.1 or S5.2=C] worst allergic reaction to peanut? Please select all the main symptoms of [your if S5=A / your child's if S5 or S5.1 or S5.2=B / the person who you look after's if S5 or S5.1 or S5.2=C] worst allergic reaction.

A	Nausea	
B	Vomiting	
C	Heartburn / bloating	
D	Stomach pain / cramps	
E	Indigestion	
F	Diarrhoea	
G	Breathing difficulties / wheezing	
H	Anxiety	
I	Tiredness (acute or sudden)	
J	Fainting, collapsing	
K	Dizziness	
L	Swelling (e.g. lips, eyes, tongue)	
M	Itching mouth / throat tightness	
N	Eczema flare / rashes	
O	Hives	
P	Itching (skin, eyes, nose)	
Q	Other (please specify) __________	

Ask if Q15=A to F

Q19. What sort of support or counselling, if any, was offered after [your if S5=A / your child's if S5 or S5.1 or S5.2=B / the person who you look after's if S5 or S5.1 or S5.2=C] (worst) allergic reaction to peanut?

A	Counselling	
B	Information about Patient Associations for peanut allergy	
C	Training on how to use emergency medication	
D	Training on what to do in an emergency	
E	Other (please specify)	
F	Do not remember	
G	None	

Emergency Medication

Q20. [Have you if S5=A / Has your child if S5 or S5.1 or S5.2=B / Has the person who you look after if S5 or S5.1 or S5.2=C] been prescribed an AAI (adrenaline/epinephrine autoinjector pen) e.g. an EpiPen for [your if S5=A / their if S5 or S5.1 or S5.2=B or C] peanut allergy?

A	Yes	
B	No	
C	Other (please specify) ___________	

If B, or C: skip to Q26

Q20.1. Who prescribed the AAI (adrenaline/epinephrine autoinjector pen) e.g. an EpiPen for [your if S5=A / their if S5 or S5.1 or S5.2=B or C] peanut allergy?

A to L	List all selected in Q13	
M	Don't know	

If A in Q20

Q21. How many AAIs (e.g. EpiPen) [have you if S5=A / has your child if S5 or S5.1 or S5.2=B / has the person who you look after if S5 or S5.1 or S5.2=C] currently been prescribed for [your if S5=A / their if S5 or S5.1 or S5.2=B or C] peanut allergy?

A	Number___________	
B	Not sure	

If A in Q20

Q22. When was the last time [you if S5=A / your child if S5 or S5.1 or S5.2=B / the person who you look after if S5 or S5.1 or S5.2=C] used [your if S5=A / their if S5 or S5.1 or S5.2=B or if S5 or S5.1 or S5.2=C] AAI (e.g. EpiPen) for peanut related allergies?

A	Less than 6 months ago	
B	In the last 6 – 12 months	
C	In the last 1 – 2 years	
D	In the last 2 to 5 years	
E	More than 5 years ago	
F	Never	

brainsell
intelligent research
undeniable truth

If A in Q20

Q23. Who showed [you if S5=A / **your child** if S5 or S5.1 or S5.2=B / **the person who you look after** if S5 or S5.1 or S5.2=C] **how to use** [**your** if S5=A / **their** if S5 or S5.1 or S5.2=B or if S5 or S5.1 or S5.2=C] **AAI (e.g. EpiPen)?**

A	Allergist / Allergy Specialist Doctor	
B	Immunologist / Immunology Specialist Doctor	
C	Pediatrician	
D	Primary Care / Family Doctor	
E	Allergy Nurse	
F	Other Nurse	
I	Pharmacist	
J	Patient Association	
K	I have never been shown how to use it	
L	Other (please specify) __________	

If A in Q20

Q24. Thinking about when [**you** if S5=A / **your child** if S5 or S5.1 or S5.2=B / **the person who you look after** if S5 or S5.1 or S5.2=C] [**were** if S5=A / **was** if S5 or S5.1 or S5.2=B or if S5 or S5.1 or S5.2=C] **prescribed** [**your** if S5=A / **their** if S5 or S5.1 or S5.2=B or if S5 or S5.1 or S5.2=C] **AAI (e.g. EpiPen), how satisfied were you with the training given about how to use it? Please indicate on a scale 1 to 5, where 1 is Completely satisfied and 5 is Not at all satisfied.**

Completely satisfied Not at all satisfied

1-------------------------- [scale of 1 to 5] ---------------------------5

[] Did not receive any training

If A in Q20; skip if did not receive any training in Q24

Q25. Thinking about when [**you** if S5=A / **your child** if S5 or S5.1 or S5.2=B / **the person who you look after** if S5 or S5.1 or S5.2=C] [**were** if S5=A / **was** if S5 or S5.1 or S5.2=B or if S5 or S5.1 or S5.2=C] **prescribed** [**your** if S5=A / **their** if S5 or S5.1 or S5.2=B or if S5 or S5.1 or S5.2=C] **AAI (e.g. EpiPen) how much training did you receive about how to use it? Please specify the approx. number of minutes of training you received**

A	Number of minutes___________	
B	Not sure	

brainsell
intelligent research
undeniable truth

Restrictions on Choice

Now we would like you to think about the impact that living with peanut allergy has on your life.

Q26. Thinking about the RESTRICTIONS you face due to [your if S5=A / **your child's** if S5 or S5.1 or S5.2=B / **the person who you look after's** if S5 or S5.1 or S5.2=B C] **peanut allergy, please indicate how restricted you feel about the choices you have to make about the following? On a scale of 1 to 5 (where 1=Not at all restricted, 2=A little, 3=Moderately, 4=Very, and 5=Extremely restricted).**

How restricted do you feel…

A: When choosing where to eat out (e.g. the places available where feel it is safe to eat)

Not at all restricted Extremely restricted
1-------------------------- [scale of 1 to 5] ---------------------------5

B: About the food you can choose when eating out (e.g. the choice of food available that is safe to eat)

Not at all restricted Extremely restricted
1-------------------------- [scale of 1 to 5] ---------------------------5

C: When choosing the shops where you can buy food (e.g. places where you feel it is safe to shop for food)

Not at all restricted Extremely restricted
1-------------------------- [scale of 1 to 5] ---------------------------5

D: When buying food from a shop (e.g. peanut free choices available when food shopping)

Not at all restricted Extremely restricted
1-------------------------- [scale of 1 to 5] ---------------------------5

E: About the choice of Nursery / Schools / University available to [you if S5=A / **your child** if S5 or S5.1 or S5.2=B / **the person who you look after** if S5 or S5.1 or S5.2=C] **to attend**

Not at all restricted Extremely restricted
1-------------------------- [scale of 1 to 5] ---------------------------5
[] Not applicable

F: About the job options available to you

Not at all restricted Extremely restricted
1-------------------------- [scale of 1 to 5] ---------------------------5
[] Not applicable

G: When socialising with friends and colleagues

Not at all restricted Extremely restricted
1-------------------------- [scale of 1 to 5] ---------------------------5

H: When going to special occasions such as birthday parties and social gatherings

Not at all restricted Extremely restricted

1-------------------------- [scale of 1 to 5] ---------------------------5

I: About the types of holiday available where you feel it is safe to go (e.g. Summer camp, backpacking, independent travel)

Not at all restricted Extremely restricted

1-------------------------- [scale of 1 to 5] ---------------------------5

J: About the choice of holiday destination venues where you feel it is safe to go to

Not at all restricted Extremely restricted

1-------------------------- [scale of 1 to 5] ---------------------------5

K: About travelling on aeroplanes

Not at all restricted Extremely restricted

1-------------------------- [scale of 1 to 5] ---------------------------5

L: When travelling on public transport (e.g. trains, buses, taxis)

Not at all restricted Extremely restricted

1-------------------------- [scale of 1 to 5] ---------------------------5

Q27. Thinking about [**your** if S5=A / **your child's** if S5 or S5.1 or S5.2=B / **the person who you look after's** if S5 or S5.1 or S5.2=C] **daily activities and routines, how much extra planning is needed so you don't worry about being exposed to peanuts? Please indicate on a scale of 1 to 5, where 1 is None at all and 5 is Very much:**

None at all Very much more

1-------------------------- [scale of 1 to 5] ---------------------------5

Q28. Thinking now about any special activities (e.g. holidays, special occasions) that would not typically be in your daily routines, how much extra planning is needed so you don't worry about being exposed to peanuts? Please indicate on a scale of 1 to 5, where 1 is None at all and 5 is Very much:

None at all Very much more

1-------------------------- [scale of 1 to 5] ---------------------------5

Q29. Now thinking about how you plan your life because of the peanut allergy, how do you rate your current quality of life on a scale of 1 to 5, where 1 is Excellent and 5 is Poor?

Excellent Poor

1-------------------------- [scale of 1 to 5] ---------------------------5

Skip if Yes in S3.1 or S6

Q30. Please briefly describe any coping strategies you have used or developed as a result of the restrictions [**you face** if S5=A / **your child faces** if S5 or S5.1 or S5.2=B / **the person who you look after faces** if S5 or S5.1 or S5.2=C] **due to the peanut allergy:**

>>skip

Coping and Managing

Q31. How satisfied are you in general with the advice given for peanut allergy by healthcare professionals? Please rate on a scale 1 to 5, where 1 is Very satisfied and 5 is Very dissatisfied.

Very satisfied Very dissatisfied

1-------------------------- [scale of 1 to 5] ---------------------------5

Q32. Have you sought information about peanut allergy from a patient group or association (e.g. via their website or helpline)?

A	Yes	
B	No	

Q33. How well would you say you cope with [**your** if S5=A / **your child's** if S5 or S5.1 or S5.2=B / **the person who you look after's** if S5 or S5.1 or S5.2=C] **peanut allergy <u>now</u> compared to when it was FIRST identified/diagnosed? Please indicate on a scale of 1 to 5, where 1 is Extremely well and 5 is Not at all well:**

A: Coping with peanut allergy NOW

Extremely well Not at all well

1-------------------------- [scale of 1 to 5] ---------------------------5

B: Coping with peanut allergy when it was FIRST identified/diagnosed

Extremely well Not at all well

1-------------------------- [scale of 1 to 5] ---------------------------5

brainsell
intelligent research
undeniable truth

Q34. How confident are you about the following? Please indicate on a scale of 1 to 5, where 1 is Extremely confident and 5 is Not at all confident:

A: Talking to new people about [your if S5=A / **your child's** if S5 or S5.1 or S5.2=B / **the person who you look after's** if S5 or S5.1 or S5.2=C] **peanut allergy**

Extremely confident Not at all confident

1-------------------------- [scale of 1 to 5] ---------------------------5

B: Recognising the symptoms of an allergic reaction in the event of accidental exposure to peanut

Extremely confident Not at all confident

1-------------------------- [scale of 1 to 5] ---------------------------5

C: Describing the symptoms of an allergic reaction to peanut to a healthcare professional

Extremely confident Not at all confident

1-------------------------- [scale of 1 to 5] ---------------------------5

D: Knowing when to use an adrenaline auto-injector

Extremely confident Not at all confident

1-------------------------- [scale of 1 to 5] ---------------------------5

E: Knowing how to use an adrenaline auto-injector

Extremely confident Not at all confident

1-------------------------- [scale of 1 to 5] ---------------------------5

Q35. How worried do you tend to be about the thought of not having access to emergency services in the event of exposure to peanut? Please indicate on a scale of 1 to 5, where 1 is Not at all worried and 5 is Extremely worried:

Not at all worried Extremely worried

1-------------------------- [scale of 1 to 5] ---------------------------5

If A in Q20

Q36. In general, how often [do you if S5=A / **does your child with peanut allergy** if S5 or S5.1 or S5.2=B / **does the person who you look after with peanut allergy** if S5 or S5.1 or S5.2=C] **carry [your** if S5=A / **their** if S5 or S5.1 or S5.2=B or if S5 or S5.1 or S5.2=C] **AAI (e.g. EpiPen) with [you** if S5=A / **them** if S5 or S5.1 or S5.2=B or if S5 or S5.1 or S5.2=C]**?**

A	Always (100% of the time)	
B	Frequently (75% of the time)	
C	Occasionally (50% of the time)	
D	Rarely (25% of the time)	
E	Very rarely (10% of the time)	
F	Never	

If A in Q20

Q37. If [**you** if S5=A / **your child** if S5 or S5.1 or S5.2=B / **the person who you look after** if S5 or S5.1 or S5.2=C] **forgot to carry** [**your** if S5=A / **their** if S5 or S5.1 or S5.2=B or if S5 or S5.1 or S5.2=C] **AAI (e.g. EpiPen), how much more or less anxious would this make you feel? Please indicate on a scale 1 to 5, where 1 is Much less anxious and 5 is Much more anxious.**

Much less anxious Much more anxious

1-------------------------- [scale of 1 to 5] ----------------------------5

Family, Friends, and Other People

Q38. Thinking about your FAMILY, please rank the following statements in terms of what you think is most true about what they believe. Please select the statements by clicking them in the order of most true to least true.

		Family
A	They believe there is too much concern over peanut allergy	
B	They make too much fuss over peanut allergy	
C	They have good awareness and understanding of peanut allergy	
D	They feel awkward when making allowances for peanut allergy	
E	They tend to be oblivious about the dangers of peanut allergy	

[] Don't know

Q39. Now thinking about your FRIENDS, please rank the following statements in terms of what you think is most true about what they believe. Please select the statements by clicking them in the order of most true to least true.

		Friends
A	They believe there is too much concern over peanut allergy	
B	They make too much fuss over peanut allergy	
C	They have good awareness and understanding of peanut allergy	
D	They feel awkward when making allowances for peanut allergy	
E	They tend to be oblivious about the dangers of peanut allergy	

[] Don't know

Q40. Now thinking about OTHER PEOPLE that you encounter in your day to day life (not family or friends), please rank the following statements in terms of what you think is most true about what they believe. Please select the statements by clicking them in the order of most true to least true.

		Other People
A	They believe there is too much concern over peanut allergy	
B	They make too much fuss over peanut allergy	
C	They have good awareness and understanding of peanut allergy	
D	They feel awkward when making allowances for peanut allergy	
E	They tend to be oblivious about the dangers of peanut allergy	

[] Don't know

Feelings and Emotions

Q41. To what extent does peanut allergy impact on your daily activities? Please indicate on a scale of 1 to 5, where 1 is Not at all and 5 is Very much:

Not at all Very much

1-------------------------- [scale of 1 to 5] ---------------------------5

Q42. How frequently are you frustrated by the limitations and restrictions of living with peanut allergy? Please indicate on a scale of 1 to 5, where 1 is Not at all and 5 is Very frequently:

Not at all Very frequently

1-------------------------- [scale of 1 to 5] ---------------------------5

Q43. Overall, how frustrated would you say you are living with peanut allergy? Please indicate on a scale of 1 to 5, where 1 is Not at all frustrated and 5 is Extremely frustrated:

Not at all frustrated Extremely frustrated

1-------------------------- [scale of 1 to 5] ---------------------------5

Q44. How would you rate the level of UNCERTAINTY of living with peanut allergy? Please indicate on a scale of 1 to 5, where 1 is Extremely low and 5 is Extremely high:

Extremely low Extremely high

1-------------------------- [scale of 1 to 5] ---------------------------5

Q45. How would you rate the level of STRESS of living with peanut allergy? Please indicate on a scale of 1 to 5, where 1 is Extremely low and 5 is Extremely high:

Extremely low Extremely high

1-------------------------- [scale of 1 to 5] ---------------------------5

Q46. Thinking about the occasions when you are away from the home environment, how WORRIED are you about exposure to peanut? Please indicate on a scale of 1 to 5, where 1 is Not at all worried and 5 is Extremely worried:

A: Social occasions where food is involved (e.g. eating out)

Not at all worried Extremely worried

1-------------------------- [scale of 1 to 5] ---------------------------5

B: Social occasions where food is not involved

Not at all worried Extremely worried

1-------------------------- [scale of 1 to 5] ---------------------------5

C: School / College / University

Not at all worried Extremely worried

1-------------------------- [scale of 1 to 5] ---------------------------5

[] Not applicable

D: Work

Not at all worried Extremely worried

1-------------------------- [scale of 1 to 5] ---------------------------5

[] Not applicable

E: Holidays

Not at all worried Extremely worried

1-------------------------- [scale of 1 to 5] ---------------------------5

F: Travel on public transport

Not at all worried Extremely worried

1-------------------------- [scale of 1 to 5] ---------------------------5

G: Going to hospital

Not at all worried Extremely worried

1-------------------------- [scale of 1 to 5] ---------------------------5

Q47. [**Have you** if S5=A / **Has your child** if S5 or S5.1 or S5.2=B / **Has the person who you look after** if S5 or S5.1 or S5.2=C] **ever been made to feel different (in a negative way) to other people because of** [**your** if S5=A / **their** if S5 or S5.1 or S5.2=B or if S5 or S5.1 or S5.2=C] **peanut allergy?**

A	Yes, very frequently	
B	Yes, quite frequently	
C	Yes, but quite rarely	
D	Yes, but very rarely	
E	No, never	

Q48. Do you think [**you have** if S5=A / **your child has** if S5 or S5.1 or S5.2=B / **the person who you look after has** if S5 or S5.1 or S5.2=C] **ever been excluded from any of the following because of** [**your** if S5=A / **their** if S5 or S5.1 or S5.2=B or if S5 or S5.1 or S5.2=C] **peanut allergy?**

		Yes	No	Not sure	N/A
A	Social occasions where food is involved (e.g. eating out)				
B	Social occasions where food is not involved (e.g. the playground, playing sports, clubs)				
C	Nursery / School / University activities				
D	Work related events / activities				
E	Group holidays and activities				

brainsell
intelligent research
undeniable truth

Q49. Have you ever experienced feelings of isolation as a result of living with peanut allergy?

A	Yes, very frequently (e.g. daily / weekly)	
B	Yes, quite frequently (e.g. monthly)	
C	Yes, but quite rarely (e.g. every few months)	
D	Yes, but very rarely (e.g. once a year or less)	
E	No, not at all	

Q50. Do you find it easy to openly discuss the impact of living with peanut allergy with family, friends, healthcare professionals, school teachers, and work colleagues?

		Yes, always	Yes, sometimes	Yes, but rarely	Yes, but very rarely	No	Not sure / NA
A	Family						
B	Friends						
C	Healthcare professionals						
D	School teachers						
E	Work colleagues						

Q51. [**Have you** if S5=A / **Has your child** if S5 or S5.1 or S5.2=B / **Has the person who you look after** if S5 or S5.1 or S5.2=C] **ever experienced any form of bullying as a result of having the peanut allergy? Please indicate on a scale of 1 to 5, where 1 is No, never and 5 is Yes, very frequently:**

No, never Yes, very frequently

1-------------------------- [scale of 1 to 5] ---------------------------5

If 2 to 5 in Q51

Q52. How would you describe the impact on you of the bullying [**you** if S5=A / **your child** if S5 or S5.1 or S5.2=B / **the person who you look after** if S5 or S5.1 or S5.2=C] [**have** if S5=A / **has** if S5 or S5.1 or S5.2=B or if S5 or S5.1 or S5.2=C] **experienced? Please indicate on a scale of 1 to 5, where 1 is Harmless and 5 is Severe:**

Harmless Severe

1-------------------------- [scale of 1 to 5] ---------------------------5

If 2 to 5 in Q51 and if S5 or S5.1 or S5.2=B or C

Q52.1. How would you describe the impact of the bullying on [**your child** if S5 or S5.1 or S5.2=B / **the person who you look after** if S5 or S5.1 or S5.2=C]**? Please indicate on a scale of 1 to 5, where 1 is Harmless and 5 is Severe:**

Harmless Severe

1-------------------------- [scale of 1 to 5] ---------------------------5

brainsell
intelligent research
undeniable truth

State/trait

The following are emotions that everyone may experience. In general, how often do you experience these feelings?' Please indicate on a scale of 1 to 5, where 1 is Never and 5 is Always

Q53. ***I feel that nothing bad will happen***

Never Always
1-------------------------- [scale of 1 to 5] ---------------------------5

Q54. ***I feel anxious***

Never Always
1-------------------------- [scale of 1 to 5] ---------------------------5

Q55. ***I feel calm***

Never Always
1-------------------------- [scale of 1 to 5] ---------------------------5

Q56. ***I can sit still easily***

Never Always
1-------------------------- [scale of 1 to 5] ---------------------------5

Q57. ***I feel tense***

Never Always
1-------------------------- [scale of 1 to 5] ---------------------------5

Cost

Now we would like you to think about the costs (time and money) associated with living with peanut allergy.

Q58. Overall, how much more or less expensive do you think it is living with a peanut allergy, compared to not living with peanut allergy. Please indicate on a scale of 1 to 5, where 1 is Much Less Expensive and 5 is Much More Expensive:

Much Less Expensive Much More Expensive
1-------------------------- [scale of 1 to 5] ---------------------------5

Q59. How would you rate the cost of the extra time required due to living with peanut allergy for planning (a) your day to day activities, and (b) any special occasions? Please indicate on a scale of 1 to 5, where 1 is Not at all significant and 5 is Extremely significant:

A: Your day to day activities

Not at all significant Extremely significant
1-------------------------- [scale of 1 to 5] ---------------------------5

B: Special occasions

Not at all significant Extremely significant
1-------------------------- [scale of 1 to 5] ---------------------------5

Final questions

Finally, there are just a few questions that will help us to group your answers:

F1. Please tell us your gender:

Gender: Female / Male

A	Female	
B	Male	
C	Prefer not to say	

F2. Please tell us the highest level of education you attained:

A	High School	
B	University	
C	Post-Graduate	
D	Professional qualifications	
E	Other	
F	Prefer not to say	

F3. Which of the following best describes your working status?

A	Employed full-time	
B	Employed part-time	
C	Unemployed / seeking work	
D	Homemaker	
E	Student	
F	Other	
G	Prefer not to say	

F4. Who do you live with in your household?

A	I live on my own	
B	Family	
C	Friend(s)	
D	With my partner	
E	Other	
F	Prefer not to say	

If F4=B or C

F5. Including yourself, how many people live in your household?

F6. Approximately, how long would it take you to reach your nearest emergency medical centre (e.g. hospital) using the fastest transport you have available?

_______hours	_______minutes

If S5=A

Thank you for taking part. Your help with this study is very much appreciated.

If S5=B or C

Your help with this study is very much appreciated and we would like to thank you for sharing your perspectives as a [parent if S5 or S5.1 or S5.2=B / **carer** if S5 or S5.1 or S5.2=C]**. We are also trying to understand the perspective of the person who has the peanut allergy and we would be extremely grateful if you could respond on their behalf. These additional questions will take approx. 10 to 15 minutes. Would you like to continue?**

A	Yes	Continue to proxy version of questionnaire
B	No	Thank and close: **Thank you for taking part. Your help with this study is very much appreciated.**

brainsell
intelligent research
undeniable truth

PROXY QUESTIONS

THE FOLLOWING TEXT IS ON **ALL SCREENS**

If S5 or S5.1 or S5.2=B

Please answer the remaining questions on behalf of your [age in S7] year old child with peanut allergy.

If S5 or S5.1 or S5.2=C

Please answer the remaining questions on behalf of the [age in S7] year old person with peanut allergy who you look after.

Poly-Allergies and other conditions

P1. In general, how important is it for [your child with peanut allergy if S5 or S5.1 or S5.2=B / the person who you look after with peanut allergy if S5 or S5.1 or S5.2=C] to be able to avoid peanuts, tree nuts, and other foods to reduce the risk of an allergic reaction? Please indicate on a scale 1 to 5, where 1 is Not at all important and 5 is Extremely important.

A: Peanut specifically

Not at all important Extremely important
1-------------------------- [scale of 1 to 5] ---------------------------5

B: Foods with a trace of peanut

Not at all important Extremely important
1-------------------------- [scale of 1 to 5] ---------------------------5

C: Tree nuts (NOT peanut)

Not at all important Extremely important
1-------------------------- [scale of 1 to 5] ---------------------------5

D: Other foods

Not at all important Extremely important
1-------------------------- [scale of 1 to 5] ---------------------------5

The remaining questions are about PEANUT allergy

Ask if Q8=A; and if S5 or S5.1 or S5.2=B or C; do not ask if Q8=B

P10. Thinking about the first time [**your child's** if S5 or S5.1 or S5.2=B / **the person who you look after's** if S5 or S5.1 or S5.2=C] **peanut allergy was diagnosed, how reassured do you think they were with the advice given? Please rate on a scale 1 to 5, where 1 is Completely reassured and 5 is Not at all reassured.**

Completely reassured Not at all reassured

1-------------------------- [scale of 1 to 5] ---------------------------5

[] I do not know

Emergency Medication

If A in Q20

P24. Thinking about when [**your child** if S5 or S5.1 or S5.2=B / **the person who you look after** if S5 or S5.1 or S5.2=C] **was prescribed their AAI (e.g. EpiPen), how satisfied were they with the training given about how to use it? Please indicate on a scale 1 to 5, where 1 is Completely satisfied and 5 is Not at all satisfied.**

Completely satisfied Not at all satisfied

1-------------------------- [scale of 1 to 5] ---------------------------5

[] Did not receive any training

If A in Q20; skip if did not receive any training in Q24

P25. Thinking about when [**your child** if S5 or S5.1 or S5.2=B / **the person who you look after** if S5 or S5.1 or S5.2=C] **was prescribed their AAI (e.g. EpiPen) how much training was given to them about how to use it? Please specify the approx. number of minutes of training given**

A	Number of minutes___________	
B	Not sure	

Restrictions on Choice

Now we would like you to think about the impact that living with peanut allergy has on [your child's if S5 or S5.1 or S5.2=B / the person who you look after's if S5 or S5.1 or S5.2=C] life.

P26. Thinking about the RESTRICTIONS [your child faces if S5 or S5.1 or S5.2=B / the person who you look after faces if S5 or S5.1 or S5.2=C] due to their peanut allergy, please indicate how restricted you think they feel about the choices they have to make about the following? On a scale of 1 to 5 (where 1=Not at all restricted, 2=A little, 3=Moderately, 4=Very, and 5=Extremely restricted).

How restricted do they feel…

A: When choosing where to eat out (e.g. the places available where they feel it is safe to eat)

Not at all restricted Extremely restricted

1-------------------------- [scale of 1 to 5] ---------------------------5

[] Don't know

[] Not applicable

B: About the food they can choose when eating out (e.g. the choice of food available that is safe to eat)

Not at all restricted Extremely restricted

1-------------------------- [scale of 1 to 5] ---------------------------5

[] Don't know

[] Not applicable

C: When choosing the shops where they can buy their food (e.g. places where they feel it is safe to shop for food)

Not at all restricted Extremely restricted

1-------------------------- [scale of 1 to 5] ---------------------------5

[] Don't know

[] Not applicable

D: When buying food from a shop (e.g. peanut free choices available when food shopping)

Not at all restricted Extremely restricted

1-------------------------- [scale of 1 to 5] ---------------------------5

[] Don't know

[] Not applicable

E: About the choice of Nursery / Schools / University available for them to attend

Not at all restricted Extremely restricted

1-------------------------- [scale of 1 to 5] ---------------------------5

[] Don't know

[] Not applicable

F: About the job options available to them

Not at all restricted Extremely restricted

1--------------------------- [scale of 1 to 5] ----------------------------5

[] Don't know

[] Not applicable

G: When socialising with friends and colleagues

Not at all restricted Extremely restricted

1--------------------------- [scale of 1 to 5] ----------------------------5

[] Don't know

[] Not applicable

H: When going to special occasions such as birthday parties and social gatherings

Not at all restricted Extremely restricted

1--------------------------- [scale of 1 to 5] ----------------------------5

[] Don't know

[] Not applicable

I: About the types of holiday available where they feel it is safe to go (e.g. Summer camp, backpacking, independent travel)

Not at all restricted Extremely restricted

1--------------------------- [scale of 1 to 5] ----------------------------5

[] Don't know

[] Not applicable

J: About the choice of holiday destination venues where they feel it is safe to go to

Not at all restricted Extremely restricted

1--------------------------- [scale of 1 to 5] ----------------------------5

[] Don't know

[] Not applicable

K: About travelling on aeroplanes

Not at all restricted Extremely restricted

1--------------------------- [scale of 1 to 5] ----------------------------5

[] Don't know

[] Not applicable

L: When travelling on public transport (e.g. trains, buses, taxis)

Not at all restricted Extremely restricted

1--------------------------- [scale of 1 to 5] ----------------------------5

[] Don't know

[] Not applicable

P27. Thinking about [your child's if S5 or S5.1 or S5.2=B / **the person who you look after's** if S5 or S5.1 or S5.2=C] **daily activities and routines, how much extra planning is needed so they don't worry about being exposed to peanuts? Please indicate on a scale of 1 to 5, where 1 is None at all and 5 is Very much:**

None at all Very much more

1-------------------------- [scale of 1 to 5] ---------------------------5

[] Don't know

P28. Thinking now about any special activities (e.g. holidays, special occasions) that would not typically be in their daily routines, how much extra planning is needed so [your child doesn't if S5 or S5.1 or S5.2=B / **the person who you look after doesn't** if S5 or S5.1 or S5.2=C] **worry about being exposed to peanuts? Please indicate on a scale of 1 to 5, where 1 is None at all and 5 is Very much:**

None at all Very much more

1-------------------------- [scale of 1 to 5] ---------------------------5

[] Don't know

P29. Now thinking about how [your child if S5 or S5.1 or S5.2=B / **the person who you look after** if S5 or S5.1 or S5.2=C] **plans their life because of the peanut allergy, how do they rate their current quality of life on a scale of 1 to 5, where 1 is Excellent and 5 is Poor?**

Excellent Poor

1-------------------------- [scale of 1 to 5] ---------------------------5

[] Don't know

Skip if Yes in S3.1 or S6

P30. Please briefly describe any coping strategies [your child if S5 or S5.1 or S5.2=B / **the person who you look after** if S5 or S5.1 or S5.2=C] **has used or developed as a result of the restrictions faced due to the peanut allergy:**

Coping and Managing

P33. How well would you say [your child if S5 or S5.1 or S5.2=B / **the person who you look after** if S5 or S5.1 or S5.2=C] **copes with peanut allergy now compared to when it was FIRST identified/diagnosed? Please indicate on a scale of 1 to 5, where 1 is Extremely well and 5 is Not at all well:**

A: Coping with peanut allergy NOW

Extremely well Not at all well

1-------------------------- [scale of 1 to 5] ---------------------------5

[] Don't know

B: Coping with peanut allergy when it was FIRST identified/diagnosed

Extremely well Not at all well

1-------------------------- [scale of 1 to 5] ---------------------------5

[] Don't know

brainsell
intelligent research
undeniable truth

P34. How confident is [your child if S5 or S5.1 or S5.2=B / the person who you look after if S5 or S5.1 or S5.2=C] about the following? Please indicate on a scale of 1 to 5, where 1 is Extremely confident and 5 is Not at all confident:

A: Talking to new people about their peanut allergy

Extremely confident Not at all confident

1-------------------------- [scale of 1 to 5] ---------------------------5

[] Don't know

B: Recognising the symptoms of an allergic reaction in the event of accidental exposure to peanut

Extremely confident Not at all confident

1-------------------------- [scale of 1 to 5] ---------------------------5

[] Don't know

C: Describing the symptoms of an allergic reaction to peanut to a healthcare professional

Extremely confident Not at all confident

1-------------------------- [scale of 1 to 5] ---------------------------5

[] Don't know

D: Knowing when to use an adrenaline auto-injector

Extremely confident Not at all confident

1-------------------------- [scale of 1 to 5] ---------------------------5

[] Don't know

E: Knowing how to use an adrenaline auto-injector

Extremely confident Not at all confident

1-------------------------- [scale of 1 to 5] ---------------------------5

[] Don't know

P35. How worried does [your child if S5 or S5.1 or S5.2=B / the person who you look after if S5 or S5.1 or S5.2=C] tend to be about the thought of not having access to emergency services in the event of exposure to peanut? Please indicate on a scale of 1 to 5, where 1 is Not at all worried and 5 is Extremely worried:

Not at all worried Extremely worried

1-------------------------- [scale of 1 to 5] ---------------------------5

[] Don't know

If A in Q20

P37. If [your child if S5 or S5.1 or S5.2=B / the person who you look after if S5 or S5.1 or S5.2=C] forgot to carry their AAI (e.g. EpiPen), how much more or less anxious would this make them feel? Please indicate on a scale 1 to 5, where 1 is Much less anxious and 5 is Much more anxious.

Much less anxious Much more anxious

1-------------------------- [scale of 1 to 5] ---------------------------5

[] Don't know

Family, Friends, and Other People

P39. Now thinking about their FRIENDS, please rank the following statements in terms of what they think is most true about what their friends believe. Please select the statements by clicking them in the order of most true to least true.

		Friends
A	They believe there is too much concern over peanut allergy	
B	They make too much fuss over peanut allergy	
C	They have good awareness and understanding of peanut allergy	
D	They feel awkward when making allowances for peanut allergy	
E	They tend to be oblivious about the dangers of peanut allergy	

[] Don't know

P40. Now thinking about OTHER PEOPLE that they encounter in their day to day life (not family or friends), please rank the following statements in terms of what they think is most true about what other people believe. Please select the statements by clicking them in the order of most true to least true.

		Other People
A	They believe there is too much concern over peanut allergy	
B	They make too much fuss over peanut allergy	
C	They have good awareness and understanding of peanut allergy	
D	They feel awkward when making allowances for peanut allergy	
E	They tend to be oblivious about the dangers of peanut allergy	

[] Don't know

Feelings and Emotions

P41. To what extent does peanut allergy impact on [**your child's** if S5 or S5.1 or S5.2=B / **the person who you look after's** if S5 or S5.1 or S5.2=C] **daily activities? Please indicate on a scale of 1 to 5, where 1 is Not at all and 5 is Very much:**

Not at all Very much

1-------------------------- [scale of 1 to 5] ---------------------------5

[] Don't know

P42. How <u>frequently</u> is [**your child** if S5 or S5.1 or S5.2=B / **the person who you look after** if S5 or S5.1 or S5.2=C] **frustrated by the limitations and restrictions of living with peanut allergy? Please indicate on a scale of 1 to 5, where 1 is Not at all and 5 is Very frequently:**

Not at all Very frequently

1-------------------------- [scale of 1 to 5] ---------------------------5

[] Don't know

brainsell
intelligent research
undeniable truth

P43. Overall, how frustrated is [your child if S5 or S5.1 or S5.2=B / the person who you look after if S5 or S5.1 or S5.2=C] living with peanut allergy? Please indicate on a scale of 1 to 5, where 1 is Not at all frustrated and 5 is Extremely frustrated:

Not at all frustrated Extremely frustrated
1-------------------------- [scale of 1 to 5] ---------------------------5
[] Don't know

P44. How would [your child if S5 or S5.1 or S5.2=B / the person who you look after if S5 or S5.1 or S5.2=C] rate the level of UNCERTAINTY of living with peanut allergy? Please indicate on a scale of 1 to 5, where 1 is Extremely low and 5 is Extremely high:

Extremely low Extremely high
1-------------------------- [scale of 1 to 5] ---------------------------5
[] Don't know

P45. How would [your child if S5 or S5.1 or S5.2=B / the person who you look after if S5 or S5.1 or S5.2=C] rate the level of STRESS of living with peanut allergy? Please indicate on a scale of 1 to 5, where 1 is Extremely low and 5 is Extremely high:

Extremely low Extremely high
1-------------------------- [scale of 1 to 5] ---------------------------5
[] Don't know

P46. Thinking about the occasions when [your child if S5 or S5.1 or S5.2=B / the person who you look after if S5 or S5.1 or S5.2=C] is away from the home environment, how WORRIED are they about exposure to peanut? Please indicate on a scale of 1 to 5, where 1 is Not at all worried and 5 is Extremely worried:

A: Social occasions where food is involved (e.g. eating out)

Not at all worried Extremely worried
1-------------------------- [scale of 1 to 5] ---------------------------5
[] Don't know
[] Not applicable

B: Social occasions where food is not involved

Not at all worried Extremely worried
1-------------------------- [scale of 1 to 5] ---------------------------5
[] Don't know
[] Not applicable

C: School / College / University

Not at all worried Extremely worried
1-------------------------- [scale of 1 to 5] ---------------------------5
[] Don't know
[] Not applicable

brainsell
intelligent research
undeniable truth

D: Work

Not at all worried Extremely worried

1-------------------------- [scale of 1 to 5] ---------------------------5

[] Don't know

[] Not applicable

E: Holidays

Not at all worried Extremely worried

1-------------------------- [scale of 1 to 5] ---------------------------5

[] Don't know

[] Not applicable

F: Travel on public transport

Not at all worried Extremely worried

1-------------------------- [scale of 1 to 5] ---------------------------5

[] Don't know

[] Not applicable

G: Going to hospital

Not at all worried Extremely worried

1-------------------------- [scale of 1 to 5] ---------------------------5

[] Don't know

[] Not applicable

P49. [**Has your child** if S5 or S5.1 or S5.2=B / **Has the person who you look after** if S5 or S5.1 or S5.2=C] **ever experienced feelings of isolation as a result of living with peanut allergy?**

A	Yes, very frequently (e.g. daily / weekly)	
B	Yes, quite frequently (e.g. monthly)	
C	Yes, but quite rarely (e.g. every few months)	
D	Yes, but very rarely (e.g. once a year or less)	
E	No, not at all	
F	Don't Know	

P50. Does [**your child** if S5 or S5.1 or S5.2=B / **the person who you look after** if S5 or S5.1 or S5.2=C] **find it easy to openly discuss the impact of living with peanut allergy with family, friends, healthcare professionals, school teachers, and work colleagues?**

		Yes, always	Yes, sometimes	Yes, but rarely	Yes, but very rarely	No	Not sure / NA
A	Family						
B	Friends						
C	Healthcare professionals						
D	School teachers						
E	Work colleagues						

Thank you for taking part. Your help with this study is very much appreciated.
